# Age-Related Alternative Splicing: Driver or Passenger in the Aging Process?

**DOI:** 10.3390/cells12242819

**Published:** 2023-12-12

**Authors:** Marco Baralle, Maurizio Romano

**Affiliations:** 1 International Centre for Genetic Engineering and Biotechnology, Padriciano 99, 34149 Trieste, Italy; barallem@icgeb.org; 2 Department of Life Sciences, University of Trieste, Via A. Valerio 28, 34127 Trieste, Italy

**Keywords:** aging, alternative splicing, senescence, age-related diseases, splicing factors, transcriptome changes, molecular aging, splice variants, splicing regulation, age-associated splicing events

## Abstract

Alternative splicing changes are closely linked to aging, though it remains unclear if they are drivers or effects. As organisms age, splicing patterns change, varying gene isoform levels and functions. These changes may contribute to aging alterations rather than just reflect declining RNA quality control. Three main splicing types—intron retention, cassette exons, and cryptic exons—play key roles in age-related complexity. These events modify protein domains and increase nonsense-mediated decay, shifting protein isoform levels and functions. This may potentially drive aging or serve as a biomarker. Fluctuations in splicing factor expression also occur with aging. Somatic mutations in splicing genes can also promote aging and age-related disease. The interplay between splicing and aging has major implications for aging biology, though differentiating correlation and causation remains challenging. Declaring a splicing factor or event as a driver requires comprehensive evaluation of the associated molecular and physiological changes. A greater understanding of how RNA splicing machinery and downstream targets are impacted by aging is essential to conclusively establish the role of splicing in driving aging, representing a promising area with key implications for understanding aging, developing novel therapeutical options, and ultimately leading to an increase in the healthy human lifespan.

## 1. Introduction

A wide range of changes in cellular mechanisms involving both transcriptional and post-transcriptional regulation have been linked to normal aging [1]. While age-related variations in the cellular environment lead to eventual molecular changes, it is also possible that the molecular changes accelerate aging and age-related disorders (ranging from hypertension to cardiovascular disease, cancer, and neurodegeneration). Furthermore, different tissues and organs may experience different age-related alterations in transcriptional and post-transcriptional regulation.

In higher eukaryotic genomes, alternative splicing (AS) of both protein and non-coding genes not only profoundly contributes to increasing the functional diversity and complexity of the whole transcriptome [2,3,4], but it also seems to be a master regulator of cellular and individual aging.

Although the majority of variations in alternative splicing events occur during development, it is estimated that approximately 30% of all alternative splicing alterations occur during aging [5,6]. As rodents and humans consistently exhibit age- and tissue-related variations in the expression of genes involved in splicing [6,7,8,9,10,11], age-related changes in splicing may be caused by the age-related decline in splicing factor expression. On the other hand, the main categories of genes with age-related altered splicing include those encoding genes with neuronal-specific activities such as synaptic transmission in the human brain [11], as well as those implicated in collagen production and post-translational modification in the human Achilles tendon [12,13]. These observations suggest that age-dependent splicing changes are more likely to occur in at least some of the same categories of tissue-specific genes that show transcriptional decline with aging.

Aging-dependent splicing alterations can explain why some genes show a tissue-specific decrease in expression. Splicing errors during pre-mRNA processing can result in the incorrect usage of alternative splice sites, leading to intron retention in the mature mRNA transcript rather than proper exon joining. Intron retention introduces premature termination codons that target the aberrant transcripts for degradation through nonsense-mediated decay (NMD). This differs from frameshift mutations caused by small insertions or deletions during splicing, which can also introduce premature stop codons but do not always trigger transcript degradation by NMD, and may allow some protein production from the altered transcripts [14,15,16]. Considering that NMD might decline with aging [17], this might affect the levels and impact of aging-related alternative splicing isoforms.

In addition, it has been observed that mutations within splice sites may activate cryptic splice sites, resulting in the erroneous processing of lamin A, a gene associated with laminopathies and implicated in premature aging and other aging-related disorders [18].

Different studies have investigated the eventual age-associated splicing changes both in different cellular models of senescence [19] and in human tissues [10,11,20]. These studies in cells have highlighted over 400 differential splicing events associated with the senescent phenotype and have found that exon skipping is the overrepresented splicing event. The studies on human tissues focused their investigation on events occurring in brain [10,11] or 48 primary tissues [20].

On the one hand, the analysis of prefrontal cortex and cerebellum tissue has found that splicing changes across a lifespan occur in over 30% of the expressed genes and that around 15% of these variations occurred between the two brain areas [10]. Over 60% of all alternative splicing events consisted of a preferential inclusion of gene segments, potentially activating nonsense-mediated decay in infants and the elderly [10].

On the other hand, the analysis of the splicing patterns in the temporal cortex of people of different ages who had frontotemporal lobar degeneration (FTLD) or Alzheimer’s disease (AD) highlighted that genes involved in mRNA processing and splicing patterns changed significantly with age and was further corroborated by studies carried out in mice [9,11].

These observations have also been supported by a recent extensive study with human samples, which highlighted approximately 50,000 splicing tissue-specific age-related events [20]. In several human tissues (including skeletal muscles, bones, skin, thymus, and white adipocytes), the transcriptomic analyses found that 0.3 to 3.2% of the examined genes exhibited AS alterations as a result of physiological aging [21].

A series of important considerations can be drawn from the observations provided in these studies. Although the sheer number of 50,000 splicing events may appear substantial, their significance relies on their functional impact. Certain splicing events may have a more notable influence on gene function compared to others, and this influence can vary among different tissues and genes. It is also essential to keep in mind that not all splicing events necessarily result in functional consequences, emphasizing the importance of a thoughtful assessment of their biological significance. Finally, the sensitivity of the method plays a crucial role in identifying which splicing events are detected. The technique’s limitations may lead to the overlooking of some events.

Overall, these studies highlight how age-related splicing events are specific to particular tissues, suggesting that aging affects different tissues in varying ways, with some tissues exhibiting a higher percentage of gene alterations due to AS events. 

This underscores the importance of understanding the tissue-specific molecular changes associated with aging and adapting interventions accordingly.

This review provides a comprehensive overview of how RNA splicing and associated factors play a crucial role in cellular senescence and neurodegeneration, and the potential avenues for future research and treatment strategies.

## 2. Age-Related Alterations in Splicing: Evidence and Observations

The multifaceted process of aging is linked to a progressive loss in physiological integrity, which causes functional decline and elevated morbidity. Aging can alter the balance of the proteins produced by a particular gene [22,23].

**Figure 1:**
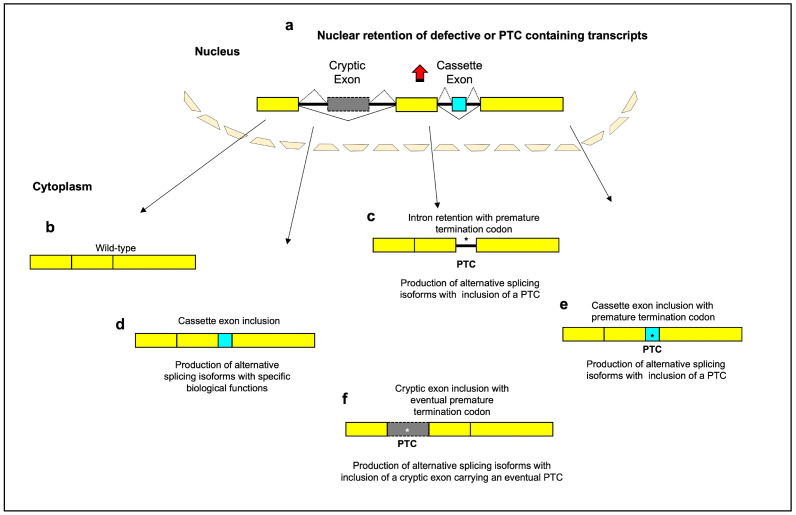
Schematic diagram of the main aging-related alternative splicing events. Most genes are split between exons and introns. Splicing-defective or premature termination codon (PTC)-containing transcripts are retained within nucleus, with inhibition of translation (**a**). Normal patterns of splice site selection (indicated with solid black lines) join the constitutive exons together to create “wild type” mRNAs (**b**). Intron-retaining transcripts can include a PTC and undergo degradation by activation of nonsense-mediated decay (**c**). The inclusion of a cassette exon into mRNA (indicated with dashed black lines) can lead to a gain of functional domains (**d**) or to the appearance of a PTC (**e**). The inclusion of a cryptic exon into mRNA can lead to the introduction of frameshifts or PTC and subsequently to the loss of specific domains or to reduction in gene expression (**f**). A portion of PTC-containing transcripts is recognized and retained in the nucleus before they have a chance to be exported to the cytoplasm for degradation by the NMD system. PTCs in different reading frames can lead to different levels of nuclear retention. This suggests that the mechanism of nuclear retention is not simply due to the presence of a stop codon, but is dependent on the specific sequence context of the PTC [30,31]. The exact mechanisms by which PTC-containing transcripts are retained in the nucleus are still being investigated. One possible mechanism is alternative splicing—the presence of a PTC can affect the splicing of an mRNA, leading to the inclusion of exons that contain nuclear retention signals that prevent export to the cytoplasm. Another possibility is interaction with specific nuclear proteins or RNA binding proteins that bind to the PTC-containing transcripts and retain them in the nucleus, blocking their export. Changes to the mRNA structure induced by the PTC could also prevent transit to the cytoplasm if the folding prevents export. While further research is needed to elucidate the precise retention mechanisms, the nuclear retention itself may serve a protective purpose. By retaining PTC-containing transcripts in the nucleus, truncated proteins are prevented from being translated in the cytoplasm where they could have detrimental effects on the cell [31,32]. Thus, nuclear retention may act more to protect the cell rather than facilitate mRNA repair pathways like splicing to excise the PTC or induce degradation of unrepairable mRNAs. Yellow box: constitutive exon; gray box: cryptic exon; aqua box: cassette exon; asterisk: premature termination codon.

This phenomenon may be considered as an evolved response to let cells change their transcriptome and proteome to adapt to the new condition. From this point of view, age-related changes in alternative splicing can contribute to and add a new degree of complexity to the control of gene expression (Figure 1). On the other hand, the age-dependent variations in alternative splicing may be considered the negative side-effect of a progressive deterioration in quality control of the splicing machinery (Figure 1), in a similar manner as has been observed at the proteomic level [24,25].

Growing evidence suggests that aging is associated with changes in splicing fidelity, primarily intron retention [26], a phenomenon conserved through evolution [26,27,28]. However, it is unclear if this is a result of aging-related disruptions in cellular homeostasis or a cause.

Intriguingly, genes involved in metabolic processes exhibit intron inclusion with age, and dietary restriction induces intron retention in young organisms like Caenorhabditis elegans [27] and Drosophila heads [26], and in the hippocampus of mice [16].

In addition, an increase in age-related intron retention in genes implicated in proteins and mRNA homeostasis has been correlated with aging-associated diseases, such as Alzheimer’s disease patients [26].

Other studies have then showed that exon skipping is prevalent in aging skeletal muscle, particularly in genes tied to mitochondrial functions and inflammation [29]. Additionally, a comparison of splicing types across tissues showed a bias towards intron retention upregulation and identified cassette exons as another frequent event [20].

These observations emphasize that age-related changes in alternative splicing contribute to the complexity of aging.

Aging is a multifaceted process associated with the progressive loss of physiological integrity, leading to functional decline and increased morbidity. These observations highlight that age-related changes in alternative splicing contribute to the complexity of the aging process. 

In conclusion, splicing changes are a dynamic and complex aspect of the aging process, involving various splicing events and tissue-specific effects, and underscore the importance of further research to elucidate the mechanisms and functional consequences of age-related splicing alterations that may provide insights into potential therapeutic strategies to mitigate age-related health issues.

## 3. Accumulation of Intron Retention Transcripts during Aging

Approximately 75% of mammal genes with multiple exons exhibit intron retention (IR) [33]. IR can induce an increase in nuclear pre-mRNA degradation by nuclear exosomes [34], an activation of nonsense-mediated mRNA decay (NMD) [35], an introduction of a mutation in the translated protein [36], or a delay in the start of gene expression through slower splicing kinetics [33].

Intron retention plays a crucial role as a negative regulator of gene expression in a variety of cellular processes, including hematopoiesis [37,38,39,40], neurogenesis [41,42], response to hypoxic stress [43], and other cellular responses [44]. Therefore, mRNA transcripts containing these retained introns are often subject to degradation through nonsense-mediated mRNA decay (NMD), eliminating those mRNA molecules that are unnecessary for the functioning of particular cell types. Nonetheless, IR can also serve as a mechanism for swiftly making specific mRNA molecules available for translation into proteins in response to particular developmental signals.

However, it is important to note that IR is also an abnormal splicing phenomenon associated with a range of disorders. A growing body of research suggests that IR is evolutionarily conserved and may play a crucial regulatory role in aging and age-related disorders [26,45,46]. Elevated IR may be a conserved post-transcriptional biomarker of aging in several species, and it is probably implicated in the pathogenesis of different neurodegenerative diseases associated with dementia [47,48,49,50,51,52]. 

Within the context of dementia pathogenesis, we want to outline the case of severe early-onset dementia resembling frontotemporal dementia associated with a specific intronic splice donor variant in the STUB1 gene [52]. STUB1 is known for heterozygous pathogenic variants causing spinocerebellar ataxia type 48 (SCA48), characterized by ataxic movement disorders and sometimes cognitive-affective symptoms. The reported case features a distinct intronic splice donor variant, c.524 + 1G > A, disrupting splicing and resulting in intron 3 retention in STUB1. This leads to the insertion of extra amino acids into the encoded protein, C-terminus of HSP70-interacting protein (CHIP). Furthermore, it triggers the activation of cryptic splice sites, with one causing a frameshift and truncation of the CHIP protein. Additionally, repeat expansions were identified in the TATA box-binding protein (TBP) gene in these patients. One patient had a heterozygous repeat expansion, while the more severely affected patient exhibited compound heterozygosity for the expansion. This case exemplifies the significance of intron retention in early-onset dementia syndromes, shedding light on its role in dementia pathogenesis.

The observation that variations in IR affect genes with different biological activities at various periods of adult life suggests that IR may have specific roles in controlling aging and animal development. 

For instance, the levels of intron retention of genes implicated in brain development, cell cycles, and epigenetic regulation are higher in young mice (2 weeks old) in comparison with older animals [26]. On the other hand, genes with a higher percentage of retained introns are engaged in controlling the equilibrium of mRNA and protein molecules in both healthy and pathological mammalian brain tissues. This would imply that the changed IR patterns may be involved in the decline of proteostasis, a key feature of normal aging [26] as well as of age-related disorders such as Alzheimer’s [26,46], ALS [51], and possibly other forms of dementia.

**Figure 2:**
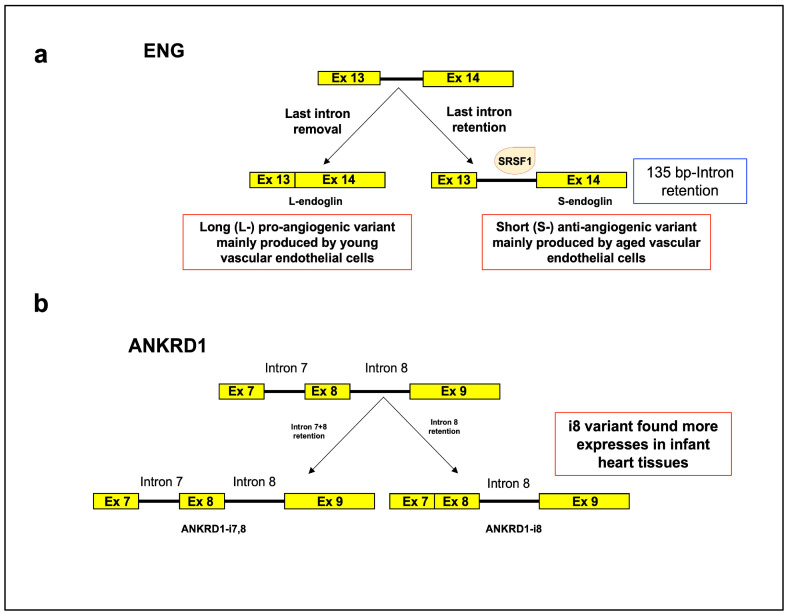
Intron retention: regulation of S-endoglin and of cardiac ankyrin repeat domain 1-i8 (ANKRD1) during senescence. (**a**) S-endoglin. During endothelial cell senescence, the splicing factor SRSF1 supports generation of the S-endoglin mRNA, by its binding to a cis-element overlapping the branch point, that hampers splicing of intron 13. As a result, SRSF1 stabilizes intron retention and increases S-endoglin mRNA levels. (**b**) Cardiac ankyrin repeat domain 1 gene (ANKRD1 or CARP). In the ANKRD1-i7,8 splicing isoform retaining both intron 7 and 8, the fourth ankyrin repeat is absent. Higher levels of the ANKRD1-i8 splicing isoform retaining intron 8 were found in infant compared to adult heart tissues. This isoform maintains all conserved ankyrin repeat motifs crucial for its interactions with titin and cardiac calsequestrin. Yellow box: constitutive exon; solid line: intron.

An example of an age-related intron retention event is the endoglin (ENG, CD105) gene, which encodes a transmembrane co-receptor for TGF-β critical for endothelial cell growth, angiogenesis, and aging (Figure 2a). Endoglin undergoes alternative splicing to generate a long (L-) pro-angiogenic variant mainly produced by young vascular endothelial cells and a short (S-) anti-angiogenic variant [53,54], characterized by the retention of a 135 bp intron between exons 13 and 14 that causes the insertion of a premature stop codon before the last exon, whose expression increases during normal aging in vitro [55,56]. During endothelial cell senescence, the splicing factor SRSF1 seems to be implicated in the generation of the S-endoglin mRNA, by interacting with a cis-element that overlaps the branch point, so generating a persistent RNA/protein complex that prevents the spliceosome from accessing the branch site. As a result, SRSF1 stabilizes intron retention and increases S-endoglin mRNA levels [56,57]. Regarding the pathological effects of this isoform, the endothelial cells from transgenic mice overexpressing S-endoglin exhibited a hypertensive response associated with a decrease in nitric oxide response inhibition [55]. 

Another example of an aging-related IR event occurs in the cardiac ankyrin repeat domain 1 gene (ANKRD1, or CARP), encoding a transcriptional cofactor in the embryonic hearts (Figure 2b). In the human heart, two intron-retaining ANKRD1 isoforms (i.e., ANKRD1-i8 and ANKRD1-i7,8) were detected, and only the i8 variant was found more expressed in infant compared to adult heart tissues, suggesting that its levels are regulated during aging [58].

Additional validated examples of intron-retaining genes are Copine 1 (CPNE1), encoding an AKT-associated calcium-dependent membrane-binding protein cascade, and Cyclin A2 (CCNA2), which encodes an essential regulator of the cell division cycle.

On the one hand, an increase in the CPNE1 mRNA species retaining the last intron (associated with lower levels of CPNE1 mRNA and protein) was observed in aged dermal fibroblasts [45]. It was suggested that this particular event was caused by the aging-dependent decreased expression of the U2AF1 splicing factor [45]. Interestingly, the splicing factor U2AF1 was also discovered to be one of the upstream factors that control phenotypes connected to senescence and global intron retention [45]. On the other hand, an increase in the cyclin A2 splice variant (called A2V6) retaining a portion of intron 6 was found only in adult, but not fetal, human tissues (in particular, heart, liver, and kidney), and this isoform was shown to cause a G1/S block in vitro and to be associated with cellular senescence [59].

Therefore, IR is a conserved process observed across a wide range of species, implying its fundamental involvement in the biology of aging. It yields various molecular outcomes, including the introduction of protein mutations, changes in gene expression, and alterations in mRNA degradation pathways, potentially contributing to the aging process and age-related disorders. IR exhibits distinct patterns in different genes and tissues, underscoring the tissue-specific nature of aging. Notably, genes associated with brain development and cell cycle regulation display unique IR patterns during the aging process.

IR may play a significant role in age-related neurodegenerative diseases, such as dementia, making it a promising target for therapeutic interventions aimed at disease prevention and management. The elevation in IR levels could potentially serve as a biomarker of aging, given its widespread occurrence across species. Monitoring IR levels could offer valuable insights into the impact of aging on overall health.

The links between IR and proteostasis, as well as cellular senescence, suggest potential associations with age-related disorders and the aging process itself. Splicing factors like SRSF1 and U2AF1 are implicated in age-related IR events, indicating their potential significance in aging biology and their potential as therapeutic targets.

In summary, the connection between IR and aging holds profound implications for understanding the fundamental biology of aging and for developing interventions that promote healthy aging and address age-related diseases.

On the other hand, while intron retention shows many intriguing associations with aging, and may exacerbate age-related cellular dysfunction, direct evidence that it can drive aging in a causal manner is currently limited.

## 4. Cassette Exons and Aging

The most frequent alternative splicing mechanism, exon skipping (ES or cassette exon) [60,61], is another splicing class of events occurring during aging. In general, ES can modify the protein function either by altering the inclusion of particular domains/sites or by introducing a frameshift of the open reading frame in the final mRNA. Indeed, the latter eventuality, along with nonsense-mediated decay (another negative impact on mRNA stability possibly caused by ES), has been recently used to “functionally” distinguish another class of alternatively spliced exons defined as “cryptic” exons (or pseudoexons) characterized by the detrimental impact of the event [60,61,62,63,64,65]. 

The first example of a cassette exon associated with aging regards SIRT1 (sirtuin 1), a member of the sirtuin family of proteins, which are NAD+-dependent deacetylases that have been linked to the regulation of aging and longevity in various organisms.

In mammals, SIRT1 expression and activity decrease with aging, and SIRT1 deficiency or inhibition leads to age-related phenotypes, such as muscle weakness, metabolic disorders, and inflammation [66]. Conversely, SIRT1 activation or overexpression has been shown to extend lifespan and delay age-related diseases in various animal models [67].

Overall, the studies support a hypothesis that the alternative splicing of human and mouse sirtuin genes can modulate the subcellular localization and function of the related protein [68]. Among the over twenty-three detected alternatively spliced human sirtuin isoforms, at least three variants seem to modify their expression during heart aging [68]. Indeed, the expression of the human SIRT1-v1 was very high in the fetal organ and then higher in 72- and 102-year-old than 24-year-old hearts. The expression of SIRT1-v2 and SIRT1-v3 variants was low in the fetal heart, it increased in 24-year-old and 72-year old hearts, and then it decreased again in 102-year-old hearts [68].

**Figure 3:**
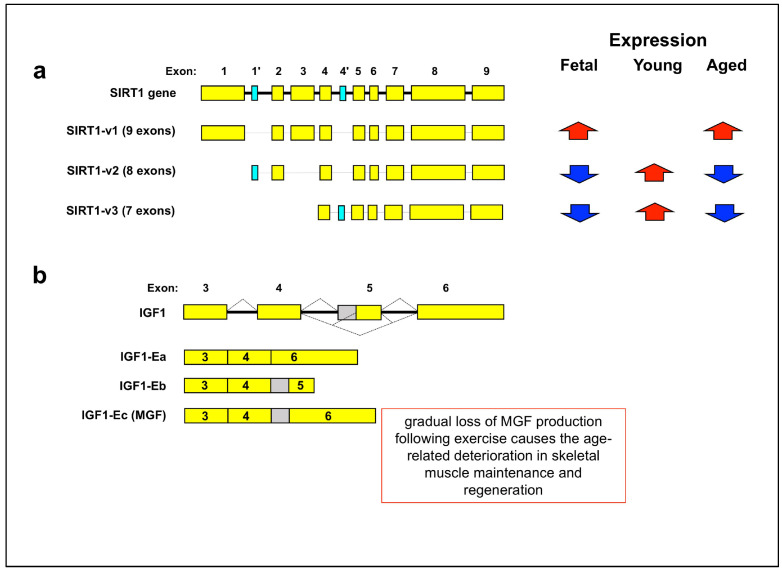
Cassette exon: aging-regulated alternative splicing of SIRT1 and ING1 genes. (**a**) The human SIRT1 genomic organization and its aging-related splicing isoforms. SIRT1-v1 isoform includes nine exons and no cassette exons (aqua coloured boxes). SIRT1-v2 includes eight exons, and the first exon was named Exon-1′ (E1′). SIRT1-v3 isoform includes seven exons with the Exon-4′ (E4′) in-between exon 4 and 5. (**b**) The human IGF1 genomic organization (IGF1) and its aging-related splicing isoforms (Ea, Eb, and Ec/MGF). The IGF1-Ea isoform shows skipping of the whole exon 5. The IGF1-Eb isoform shown includes the whole exon 5. The Ec/MGF isoform contains the N-terminal part of exon 5 (consisting of 49 nucleotides, shown as a gray box) and exon 6. Exon 5 inclusion causes the occurrence of an altered C-terminal amino acid sequence as a result of a shift in the reading frame. Yellow box: constitutive exon; solid line: intron; dotted lines: alternative splicing patterns; upwards red arrow: upregulated expression; downwards blue arrow: downregulated expression.

SIRT1-v2 and SIRT1-v3 isoforms show both exon skipping and inclusion events. In fact, in the SIRT1-v2 isoform (Figure 3a), there is a skipping of exon-1 and exon-3, as well as an inclusion of an extra exon (exon-1′ or ex1′), used as its first exon. In the SIRT1-v3 isoform (Figure 3a), there is a skipping of exon-1, -2, and -3, as well as an inclusion of an extra exon (ex4′). Interestingly, the intracellular distribution of the isoform proteins seems to be different (nuclear for v1, cytoplasmatic for v2 and v3) depending on the presence of a nuclear localization signal sequence within exon-1 and exon-3 [68]. In addition, since the alternative splicing events in SIRT1-v2 and -v3 isoforms cause a shortening of the N-terminal region, which is critical for protein–protein interaction and deacetylation [68], these events reflect the complex and still-unclear functional changes of SIRT1 during organ aging.

Another example of the aging-associated cassette exon regards the Insulin Growth Factor-1 (IGF-1) whose production and signaling are reduced by caloric restriction, a state that lengthens life in various species. The human IGF-1 gene contains six exons and undergoes alternative splicing with the generation of three different isoforms: IGF-1Ea, IGF-1Eb, and IGF-1Ec [69,70,71,72]. IGF-1Ea is the most common variant and lacks exon 5, while IGF-1Ec, also known as the Mechano Growth Factor (MGF), contains part of exon 5 joined to 6. The consequence of this latter splicing event, whose generation is mediated by the recruitment of SRSF1 to the IGF-1 gene’s exon 5 by a regulatory ESE [73], is a frame-shift resulting in the translation of an isoform with a C-terminus carrying only a portion of the functional E-domain [71,74,75,76]. The third isoform, IGF-1Eb, lacks exon 6, and carries only the first 17 amino acids identical to those in the MGF isoform [77]. In general, the main form, IGF-1Ea, is expressed in the liver and is a circulating factor. On the other hand, IGF-1Eb and MGF show a tissue-specific pattern of expression in response to different pathological events [78,79,80]. 

The human MGF isoform was discovered to greatly prolong the proliferative life span and postpone the senescence of satellite cells derived from neonatal and young adult muscle, but not from old adult muscle [75]. The potential relevance of the MGF isoform in the aging context is further supported by a number of observations made in mice, rats, and humans. On the one hand, its expression in neurogenic areas of the murine brain drops with aging, and its overexpression raises the number of neural progenitor cells without altering the post-mitotic distribution of adult newborn neurons [81]. On the other hand, exercise seemed to increase MGF levels in young but not older rats as well as in people [82,83], and this lends support to the hypothesis that a gradual loss of MGF production following exercise causes the age-related deterioration in skeletal muscle maintenance and regeneration [84].

The Inhibitor Of Growth Family Member 1 (ING1) gene encodes a nuclear factor that interacts with the tumor suppressor protein TP53 [85], blocks cell growth, and induces apoptosis [86].

Among the described ING1 splicing variants [87], two cassette exon isoforms, which encode the p47ING1a (ING1v4 deriving from Exon 1b–Exon 2 splicing) and p33ING1b (ING1v1 deriving from Exon 1a–Exon 2 splicing) proteins, show age-dependent apoptotic properties [88]. 

In fact, in senescent fibroblasts, there is a significant shift in the relative expression levels of the two main ING1 isoforms, ING1a and ING1b, with the INGla protein being expressed at greater levels and the INGlb protein being produced at lower levels. Due to this differential expression (although the variations in protein levels did not precisely correspond to the variations in mRNA expression levels), the INGla:INGlb mRNA ratio gradually changes by a factor of approximately 30-fold as compared to young fibroblasts. Indeed, the ING1a isoform overexpression in fibroblasts promotes cell cycle arrest and causes senescence-related alterations such as cell flattening and increases in heterochromatin foci as well as in beta-galactosidase activity [89]. Subsequently, it was shown that the p53-dependent ability of INGla to induce senescence is probably a mechanism of tumor protection [90] and relies on its ability to bind the chromatin mark H3K4me3 and to attract histone-modifying complexes [90,91]. This latter observation opens an intriguing possible connection between the histone code, cassette exons, and aging. On the one hand, recent studies have shown that epigenome-level histone alterations have a role in controlling RNA splicing. For instance, it has been found that H3K9me2 and H3K27me3 control human Fibronectin (FN1) gene exon EDI inclusion, H3K36me3 controls the alternative splicing of the FGFR2 (Fibroblast Growth Factor Receptor 2) exon IIIb [92], and other cassette exon events are controlled by the combinatorial effect of histone modifications [93]. Considering that at least FN1 EDA splicing seems to be aging-regulated, these observations suggest that deciphering the splicing code from histone changes will open up new avenues for understanding mechanisms underlying aging-related cassette exon (and not only) splicing regulation.

Thus, altogether, these observations regarding the association between aging and exon skipping highlight the intricate relationship between alternative splicing and the aging process. However, more research is needed to conclusively demonstrate whether it can directly cause or drive aging changes, rather than just being associated with or exacerbated by aging.

## 5. Sexually Dimorphic Alternative Splicing Signatures in Aging (Does Sex Make a Difference in Alternative Splicing in Aging?)

Sex-related differences in splicing [94,95] and diseases [96,97] are widely documented. These can be influenced by genetics, hormones, lifestyle choices, and social factors [98,99,100,101,102].

A study examining gene activity across 12 brain regions in 137 postmortem samples found sex differences in gene expression and splicing in all areas, affecting approximately 2.5% of expressed genes [95]. Some differentially expressed genes important for diseases showed sex-biased patterns, suggesting implications for sex differences in disease susceptibility. 

Interestingly, this study found 448 genes with sex-biased expression in the brain, with 85% displaying sex-biased splicing. Notably, the observation that 95% of the genes with sex-biased splicing were on autosomes rather than sex chromosomes suggested that sex-biased gene expression and splicing may contribute to sex differences in disease, independently of X chromosome effects. A correlation between sex-biased alternative splicing and aging and different studies have shown that a significant number of genes exhibit differential splicing patterns between males and females in various tissues as they age [20,103,104,105].

These observations reveal that sex-specific differences extend beyond gene expression to molecular processes like splicing. Furthermore, these sex-biased splicing events can influence the expression of genes that are relevant to various diseases. This implies that the differences in alternative splicing between males and females may play a role in the susceptibility to these diseases and may have broader implications for health.

These findings underscore the need for extensive research into the molecular mechanisms underpinning sex-related alternative splicing. Investigating how hormones, genetics, and lifestyle factors influence splicing events can provide a deeper understanding of the complex interplay between sex and splicing.

Recognizing the existence of sex-related differences in alternative splicing is essential for tailoring healthcare approaches to individual needs. Understanding how splicing patterns vary between sexes can lead to more effective diagnostic and treatment strategies, especially for diseases with sex-related differences in susceptibility.

## 6. The Emergence of Cryptic Exons in Aging and Disease

Another sign of age-related “splicing damage” is the activation of cryptic exons (also known as “pseudoexons”), consisting of splicing variants eventually activated by mutations creating new splice sites, or the removal of the existing binding sites for splicing inhibitors, as well as by a decrease in the levels of the repressor splicing factors. The functional consequence of the cryptic exon activation is the introduction of frameshifts or premature translation stop codons predicted to induce nonsense-mediated decay [62,106,107,108,109,110,111,112] or a loss of function phenotype. 

In this context, probably the most popular example of cryptic exon activation associated with aging regards the Lamin A (LMNA) gene. The premature aging Hutchinson-Gilford progeria syndrome (HGPS or Progeria) is characterized by the occurrence of a C-to-T silent mutation (LMNA c.1824 C > T, p.Gly608Gly) in exon 11 of the LMNA gene [113], encoding for an intermediate filament protein critical for maintenance of the nuclear envelope. The c.1824 C > T substitution leads to an aberrant splicing and usage of a site within the normal exonic sequence. This alternative splicing event causes a frameshift leading to the production of a truncated splice isoform (Δ50 aa, called progerin) that, by acting as a dominant factor, causes numerous morphological anomalies of cell nuclei and impairs heterochromatin organization [114]. 

It was found that the wild-type progerin 5′ donor splice site forms a stable RNA structure that hampers its pairing with U1 snRNA [115]. The progeria mutation weakens this structure, making the cryptic 5′ splice site more accessible to the splicing machinery. Moreover, in wild-type cells, the cooperation of the SRSF1, SRSF6 [115], and possibly SRSF5 [116] splicing factors promotes lamin A donor splice site (rather than progerin) selection. Apparently, both mechanisms contribute to inhibit the generation of progerin during normal aging. 

Interestingly, the accumulation of prelamin A, an unprocessed precursor of lamin A containing a C-terminal extension, has been correlated with normal aging. While this observation suggests that prelamin A could contribute to driving aspects of the aging process, further evidence is needed to conclusively establish a causal role [114,117].

Another example of cryptic exon activation associated with senescence and aging is the p53β isoform, produced by retention of a cryptic exon within intron 9 of the p53 gene via alternative splicing [118]. It was found that the splicing factor SRSF3 normally hampers the activation of cryptic exon 9 in proliferating normal human fibroblasts [119], and a decrease in its levels during aging results in inclusion of the cryptic exon in the final mRNA, with the introduction of a premature stop codon that causes the insertion of a short 10-residues-tail in place of the full-length C-terminal oligomerization domain. Indeed, p53β can induce cellular senescence by upregulating particular p53-target genes, such as p21Waf1/Cip1 [119].

Other examples of cryptic exon activation associated with age-associated diseases were found during investigations on amyotrophic lateral sclerosis (ALS) and frontotemporal lobar degeneration (FTD), as well as in Alzheimer’ disease (AD), and at least some of them were related also with aging. 

In this regard, the abnormal TDP-43 nuclear clearance initially observed in ALS and FTD [120] was also detected in AD [121]. Although cryptic exon inclusion likely involves multiple factors beyond just the loss of one repressor, the fact that TDP-43 functions as a splicing repressor of cryptic exons and that this function is impaired in the brains of ALS and FTD patients leads to the hypothesis that the nuclear clearance of TDP-43 has a crucial role in suppressing cryptic exons. Interestingly, TDP-43 is the first member of a special class of microsatellite binding cryptic exon repressors (which also includes the polypyrimidine track-binding proteins 1 (PTBP1) and 2 (PTBP2), able to repress cryptic exons by utilizing CU dinucleotide repeats [122], whose expression could also undergo a physiological decrease during aging [123,124].

**Figure 4:**
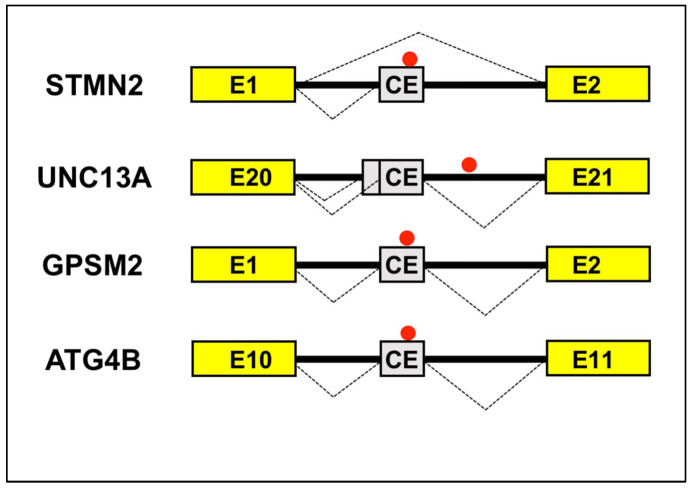
Aging-related cryptic exons. Schematic representation of cryptic exons activated by loss of TDP-43. For UNC13A gene, two alternative acceptor splice sites can be activated, causing the insertion of 128 bp or 178 bp cryptic exons. The exons immediately upstream and downstream of the cryptic exons are shown with the corresponding number. Yellow box: constitutive exon; solid line: intron; dotted lines: alternative splicing patterns; gray box: cryptic exon (CE); red point: the position of the putative TDP-43 binding site within the in-between intron.

The list of cryptic exons regulated by TDP-43 is growing [111,120,125]. Among those which seem to show an association with aging, Stathmin-2 (STMN2), a microtubule-associated protein implicated in axonal regeneration [126], was discovered to be dependent on TDP-43 for proper splicing and expression [127,128]. In fact, it was found that TDP-43 binds to GU-rich regions in the first intron of the STMN2 mRNA (Figure 4), so inhibiting the use of an alternate or cryptic polyadenylation site and consequently enabling regular intron 1 splicing. Following TDP-43 nuclear depletion, the cryptic exon (named exon 2a) is included in STMN2 mRNA, causing the insertion of a premature stop codon and the accumulation of a truncated STMN2 variant that lacks exons 2 through 5.

Another example of this class of events is UNC13A, a gene encoding a neuronal protein critical for the vesicle priming phase (occurring before synaptic vesicle fusion) [129,130], whose deficiency in mice results in an early death [131,132].

Whereas human UNC13A does not normally include the cryptic exon, in the ALS and FTLD patients’ neurons, it has been found to be one of the genes with the most significant levels of alternative splicing, with the insertion of a 128 bp or 178 bp cryptic exon (Figure 4) between canonical exons 20 and 21, as a result of the loss of TDP-43 [125,133]. Similar to what has been observed in the STMN2 gene, the UNC13A cryptic exon also contains a stop codon, which is supposed to cause nonsense-mediated decay of the mRNA.

Interestingly, 25% to 50% of Alzheimer’s cases include TDP-43 pathology, particularly those with a more severe clinical phenotype, and those showing hippocampal sclerosis (HS) [134].

In this regard, it was hypothesized that the accelerated cognitive deterioration in AD cases with TDP-43 disease may be caused by a loss of TDP-43’s capacity to suppress cryptic exons. A recent study found the incorporation of known TDP-43-regulated cryptic exons of G Protein Signaling Modulator 2 (GPSM2, within intron 1, Figure 4), and autophagy-related 4B cysteine peptidase (ATG4B, within intron 10, Figure 4) genes [121]. Also in these cases, the insertion of the cryptic exon causes the occurrence of a PTC.

In summary, the emergence of cryptic exons in aging and age-related diseases suggests that splicing events play a significant role in both the aging process and age-related disorders.

The activation of cryptic exons during aging and age-related diseases suggests a connection between these splicing events and the aging process. Understanding the factors and mechanisms contributing to the activation of cryptic exons may provide insights into the molecular basis of aging and age-associated disorders.

The example of progerin and its role in both HGPS and normal aging implies that cryptic exon activation could play a role in natural aging processes. This highlights the importance of studying cryptic exons not only in rare genetic conditions but also in the context of general aging and age-related diseases.

The involvement of splicing factors like TDP-43 in regulating cryptic exons and their potential decline during aging raises questions about the interplay between splicing and the aging process. Investigating the role of splicing factors in age-related cryptic exon activation may lead to a better understanding of the molecular mechanisms underlying aging.

## 7. Mining Databases for Splicing Factors Linked to Human Aging

Various available resources currently aim to support research on the genetics of human aging [135,136,137,138,139]. We probed the CellAge database, a repository of genes linked to cell senescence [138,139], for the presence of genes categorized under Gene Ontology ID 0008380 (specifically related to RNA splicing). Our investigation revealed an overlap of 50 genes with the list of genes that either show increased or decreased expression during replicative senescence of human cells (Table 1).

The intersection of splicing-related genes with cellular senescence presents a compelling connection, suggesting that the splicing machinery and the intricate regulation of gene expression through splicing may have a significant role in the senescence process. This association underscores the potential influence of RNA processing on the mechanisms governing aging and cellular senescence.

However, it is crucial to recognize that the interplay between genes and biological processes is far from straightforward. The complexity of these relationships is exemplified in this analysis. Splicing-related genes are not uniform in their senescence-related effects; rather, they exhibit a range of roles. Some of these genes may act as activators of senescence, while others serve as inhibitors, indicating a nuanced interplay within the cellular context. The majority of these genes display under-expression during replicative senescence. This phenomenon suggests that these genes are subject to downregulation as cells progress towards replicative senescence, aligning with the established notion that changes in gene expression are a hallmark of cellular senescence.

Remarkably, the analysis also brought to light the presence of two genes, AHNAK2 and RBM24, which buck the trend by being over-expressed during replicative senescence. This discovery is particularly intriguing as it emphasizes that while downregulation of splicing-related genes is a common theme, there exist specific genes that undergo an increase in expression as senescence sets in. As such, further exploration of the roles played by AHNAK2 and RBM24 in senescence holds the promise of shedding light on the underlying molecular mechanisms.

The observation that splicing-related genes exhibit differential expression during senescence further substantiates the connection between splicing and the senescence process. This connection is reinforced by the understanding that alternative splicing is a pivotal step in the regulation of gene expression. Dysregulation of this process can have far-reaching effects, potentially impacting the transcriptome and proteome, and in turn, influencing pathways linked to senescence.

The intriguing aspect of a significant number of splicing-related genes experiencing under-expression during replicative senescence hints at the possibility of a broad-scale suppression of splicing machinery. This suppression could, in turn, impact the processing of pre-mRNA and the synthesis of functional proteins, ultimately leading to altered cellular functions and contributing to the senescence phenotype.

On the other hand, the analysis categorically outlines the associations of identified splicing-related genes with senescence induction or inhibition. Notably, HNRNPA1, HNRNPA3, and NPM1 are under-expressed and are associated with the inhibition of senescence. In contrast, hnRNPC stands out as the only splicing-related factor associated with senescence induction, despite being under-expressed. These observations underscore the intricacy of cellular senescence and emphasize the multifaceted roles of various genes in the regulation of this process. They underscore the notion that senescence is a delicately balanced phenomenon influenced by numerous genetic and molecular factors.

Understanding the roles of these splicing factors in senescence opens the door to potential clinical implications. Notably, if specific factors are linked to senescence induction and possess established roles in the process, they might be considered as viable targets for interventions aimed at modulating senescence. Such interventions hold promise in addressing aging-related conditions and age-related diseases, making this field of research highly relevant and potentially transformative.

## 8. Pathophysiologic Correlations between Upstream Regulators and Age-Related Alternative Splicing Variations

Several longitudinal studies investigated eventual aging-related changes in the expression of splicing factors in multiple tissues (brain, blood, liver, skin, muscle) as well as in different organisms (human, mouse, fruit fly and worms), without outlining, in most cases, any single or set of “driver” splicing factors altered during aging across all tissues and species [9,10,11,124,140,141].

Indeed, a study on primary human cells aged to senescence in vitro reported that changes in tissue-specific splicing factor expression during aging may be partially attributable to age-related alterations in Ataxia Telangiectasia Mutated (ATM) gene expression [6]. The ATM gene is a key regulator of the complex DNA damage response signaling pathway and has been proposed as a potential modulator of splicing factor levels. Specifically, this study found that ATM knockdown correlated with the altered expression of seven splicing factors—SRSF1, SRSF2, SRSF3, SRSF7, TRA2B, hnRNPA1, and hnRNPD [6].

Interestingly, ATM levels seem to correlate differently with age depending on cell type. In leukocytes from a population study, ATM levels were inversely associated with age. However, senescent fibroblasts showed a positive correlation between ATM levels and age [6]. 

These aberrant splicing patterns likely contribute to the loss of cellular adaptability and plasticity during aging. They could also have downstream impacts like the altered proportions and effectiveness of innate and adaptive immune cells. This may explain the alteration of immune responses and enhanced susceptibility to infections observed in the elderly [142,143,144].

Conversely, the heterogeneity of these results can be ascribed, at least partially, to the variability in biological sampling and cellular composition of the samples as well as in the relative aging rate of the tissues analyzed in the different studies. Also, differences in the technologies and bioinformatic analysis used across the studies can lead to the same determination of low homogeneity, also taking into consideration that the changes in mRNA level do not always mirror those at the protein level.

In general, what emerges is a large variability among studies, with a general age-related downregulation of expression of splicing factors in the brain, blood, liver, and skin compared to the upregulation of expression of splicing factors in muscles. It is also interesting to notice that alternative splicing alterations are also observed in multiple tissues across various species during aging, except in animals with negligible senescence [7,20,21,23].

Among the splicing factors, SF3B1, SRSF1, SRSF2, SRSF5, SRSF6, NOVA1, HNRNPA0, HNRNPD, HNRNPK, HNRNPH1, HNRNPH3, HNRNPM, and TDP-43 are those whose expression was found to change in different tissues and organisms [9,10,11,124,140,141]. 

In addition, the experimental induction of defects in RNA processing is associated with accelerated aging. Importantly, obtaining complete knockout data for many splicing factors is challenging due to their lethality or induction of severe fetal growth alterations [145,146]. On the other hand, studies focused on single splicing factors have shown that hnRNPD (AUF1) deficiency in mice results in telomere attrition that drives accelerated aging that can be corrected by reconstituting hnRNPD/AUF1 expression, highlighting its key role in telomere maintenance and aging [147], along with cellular senescence resulting from the depletion of HNRNPD or SRSF2 expression [148]. Also, the alteration of HNRNPA3, SRSF7, and SRSF4 expression levels, as well as the disruption of transcriptome-wide splicing patterns using a pharmacological inhibitor of SF3B1, is also associated with the induction of senescence [149]. On the other hand, the restoration of splicing factor expression levels has been shown to rescue various aspects of the senescent cell phenotype [150,151,152]. In fact, not only was overexpression of the RNA processing factor PRP19 able to extend the lifespan of human endothelial cells in vitro by enhancing stress resilience and DNA repair capacity [150], but it has also demonstrated the ability to increase lifespan in a Drosophila systemic model [153].

It is interesting to notice that the mechanisms controlling the age-related changes in the expression levels of splicing factors are still largely unknown. It was hypothesized that the observed age-related variations might be caused by transcriptional, signaling, and epigenetic alterations, as well as by somatic mutations.

In this latter regard, the occurrence of mutations in core spliceosome genes that increase the risk of age-related diseases further support the critical role of alternative splicing for aging. In fact, mutations in U2AF1, SF3B1, and SRSF2 genes are frequently found in patients affected by myelodysplastic syndromes (MDSs), which are myeloid cancers typical of the elderly [154,155,156]. Approximately 9% of people with MDS present with a missense mutation (S34F) in the splicing factor U2AF1, which, in transgenic mice, showed an ability to alter splicing in hematopoietic progenitor cells and to cause defective hematopoiesis [157,158,159]. 

Then, mutations were found also in the spliceosome genes SF3B1 and SRSF2 of 42% of MDS patients, and these splicing factors were significantly linked to the various clinical manifestations of MDS [154,155,160]. Importantly, mutations in SF3B1 and SRSF2 were also exclusively found in individuals over 70 years old, supporting the high prevalence of clonal hemopoiesis linked to these mutations in old age [154,156].

Finally, the interplay between aging and mutations in causing age-related symptoms is not fully understood, especially for nuclear factors like TDP-43 involved in RNA metabolism [161], which are implicated in FTD and other neurodegenerative disorders. Typically, the effects of mutations manifest later in life, pointing to the role of age-dependent decline in cellular quality control [162]. Aging changes the cellular environment in specific cell types, possibly altering the RNA-binding protein ecosystem and causing dysfunction [163,164]. Additionally, stochastic aging processes may increase the impact of some proteins and accelerate oxidative and DNA damage buildup [165]. These gaps highlight the challenge in fully grasping the role of alternative splicing in the pathogenesis of age-linked diseases.

A deep dive into longitudinal studies across various tissues and organisms reveals the complexity of age-related alterations in the expression of splicing factors. While these studies do not consistently pinpoint specific “driver” splicing factors altered during aging across all tissues and species, they do shed light on several noteworthy observations.

These age-related splicing alterations likely contribute to the decline in cellular adaptability and plasticity associated with aging. The resulting changes in the proportions and effectiveness of innate and adaptive immune cells could help explain the increased susceptibility to infections observed in the elderly.

Moreover, variations in technologies and bioinformatic analyses used across studies can further contribute to the observed diversity. It is important to note that changes at the mRNA level may not always mirror those at the protein level.

On the other hand, some implications emerge from these findings. Splicing factors that show consistent alterations in expression across various tissues and organisms, such as SF3B1, SRSF1, SRSF2, and others, could serve as potential therapeutic targets for age-related diseases (i.e., modulating the expression or activity of these factors may help mitigate the effects of aging).

The age-related changes in alternative splicing patterns significantly impact cellular functions and contribute to the aging process. Understanding these changes is critical for uncovering potential interventions to counteract age-related functional decline and diseases.

Mutations in core spliceosome genes, such as U2AF1, SF3B1, and SRSF2, are found at higher rates in older individuals and underscore the role of alternative splicing in age-related disorders.

The interplay between aging and mutations in causing age-related symptoms is not fully understood. However, it is clear that aging alters the cellular environment, possibly impacting the RNA-binding protein ecosystem and leading to dysfunction. Understanding these age-dependent changes in quality control mechanisms is essential for elucidating the pathogenesis of age-linked diseases.

The ability to rescue senescent cell phenotypes through the restoration of splicing factor expression, as demonstrated with PRP19, suggests a translational potential for splicing-factor-based interventions to extend the healthspan and lifespan.

## 9. Is Aging-Related Alternative Splicing a Feasible Druggable Target?

The possibility of treating illnesses or aging-associated phenotypes by mRNA mis-splicing correction is a compelling opportunity. Table 2 summarizes the examples of possible anti-aging treatments described in the text. Among the currently possible therapeutic targets, the LMNA C-to-T silent mutation (p.Gly608Gly) has been successfully targeted by using antisense oligonucleotides (ASOs) both in fibroblasts from HGPS patients and in a mouse model of human HGPS, where the progerin levels and nuclear abnormalities have been rescued by treatment with antisense morpholinos oligos [166,167,168]. On the other hand, considering that a number of splicing factors implicated in the regulation of aging-related alternative splicing can be mutated and cause aging-related diseases such as familial and sporadic amyotrophic lateral sclerosis (ALS) and frontotemporal dementia (FTD) [161,169,170], the targeting of these mutated factors might represent an alternative therapeutic strategy. In this regard, studies carried out in human patients cells and in mice producing the hexanucleotide expanded C9ORF72 RNAs (causing familial and sporadic amyotrophic lateral sclerosis—ALS—and frontotemporal dementia—FTD) have shown that the treatment with ASOs selectively targeting hexanucleotide GGGGCC repeats is able to decrease the levels of repeat-containing C9ORF72 RNA variants (while maintaining levels of wild-type C9ORF72-encoding mRNA), and to relieve age-dependent behavioral deficits in mice [171,172,173]. Moreover, another lesson received from studies on cancer is that the mutated splicing factors might exhibit a different sensitivity to some drugs, compared with wild-type isoforms. In fact, an increased sensitivity to spliceosome inhibitors has been reported in a murine leukemia model carrying the MLL-AF9 fusion oncogene; in the splicing factor Srsf2 P95H, which was found to be more sensitive to E7107 (small-molecule spliceosome inhibitor, semisynthetic derivative of pladienolide B) than Srsf2+/+ wild-type leukemias [174]; as well as in Sf3b1 K700E/+ and U2af1 S34F/+ murine hematopoietic cells in vivo [175,176] and in an SRSF2-mutant CMML-patient-derived xenograft model [177]. 

Another study investigated the inhibitory effects of Spliceostatin A (SSA), a methylated derivative of FR901464, on mRNA splicing and its potential impact on hematopoietic stem/progenitor cell (HSPC) aging [178]. Treatment with SSA induced notable manifestations of cell senescence in HSPCs, as evidenced by an increase in SA-β-gal stain-positive cells and a reduction in the mixed colony-forming unit (CFU-Mix) of HSPC culture. These results suggested that the inhibition of mRNA splicing by SSA may contribute to HSPC aging, highlighting mRNA splicing as a crucial mechanism in the aging process.

These findings hold implications for potential therapeutic strategies aimed at mitigating the effects of aging and then highlight the importance of drug screenings to find novel splicing inhibitors that might directly target the mutated splicing factors whose effects might be helpful to rescue the aging-related splicing defects.

Finally, in the last few years, the exosome has gained attention for its implication in RNA degradation [179]. 

The complex biology of aging stems from diverse molecular pathways and is unlikely to be addressed through a singular target or intervention. However, the exosome’s role in degrading intron-retaining transcripts highlights its potential as a component of combinatorial therapies to promote healthy longevity. By removing senescent cells and RNAs that accumulate during aging, exosome-modulating senolytic drugs could help counteract certain age-related phenotypes. Yet, given the multifaceted nature of aging, exosome-based drugs would likely need to be part of an integrated therapeutic strategy. While treatments aimed at selectively clearing senescent cells represent exciting frontiers in geroscience, fully addressing the intricate biology of aging will require nuanced, multipronged approaches that account for its systemic complexity.

In conclusion, the pathogenesis of age-related diseases is multifactorial, stemming from complex interactions between diverse genetic and environmental factors. While alterations in splicing likely contribute to disease development in some contexts, they represent just one piece of an intricate molecular puzzle rather than the sole driver. Efforts to understand and treat age-related diseases must recognize splicing changes as just one part of the multifaceted pathogenic processes driven by complex interactions between genetics, the environment, tissue contexts, and stochastic factors. Probably, therapeutic strategies will require targeting not just splicing defects but the diverse landscape of molecular alterations underlying these disorders.

Therefore, by leveraging cutting-edge technologies, these obstacles might be overcome, and, in such a way, it might potentially pave the way for the development of novel combined therapies that effectively target the root causes of aging.

Table 2 presents a comprehensive overview of the impact of various compounds and molecules on splicing events, splicing factors, and aging. The selection of these compounds is based on their use in regulating aging-related splicing events. The table provides insights into the diverse mechanisms employed by these molecules and their potential implications for splicing regulation and aging.

## 10. Conclusions

The connection between alternative splicing and aging is a captivating yet intricate field within our comprehension of the aging process. This intricate interplay encompasses alterations in transcription, signaling, epigenetics, and somatic mutations.

Assessing alternative splicing patterns, especially in critical genes or tissues, has the potential to serve as valuable indicators of the aging process. This may yield valuable insights into how aging impacts an individual’s health and susceptibility to age-related diseases.

From this perspective, gaining a more profound comprehension of the role of alternative splicing in aging could uncover potential targets for therapeutic interventions aimed at enhancing healthy aging and addressing age-related diseases.

Although a strong association between alternative splicing events and aging exists, differentiating between correlation and causation remains a formidable challenge. 

Observing and considering a splicing factor or a splicing event as a driver of the aging process involves a comprehensive evaluation that encompasses various aspects of molecular and physiological changes associated with aging. Different key factors and criteria should be considered, as summarized in Table 3.

Considering these elements, the alternative splicing event related to the lamin A gene, leading to progerin production in HGPS, can indeed be considered a driver of aging, especially in the context of this specific accelerated aging disorder.

Regarding other cases of alternative splicing related to aging, existing evidence suggests that changes in splicing may indeed contribute to various aspects of the aging phenotype. Nonetheless, none of these cases satisfy all the criteria completely. Hence, further research is necessary to conclusively substantiate these associations as drivers of aging.

Consequently, the intricate interplay between alternative splicing and aging remains a promising realm for exploration, holding significant implications for our understanding of the biology of aging.

## Figures and Tables

**Table 1:** Overlap between the genes categorized under Gene Ontology ID 0008380 and the list of genes that either show increased or decreased expression during replicative senescence of human cells available in the CellAge Database (https://genomics.senescence.info/cells/, accessed on 11 December 2023). The table presents the results of matching the 1159 human orthologs of RNA binding proteins categorized under Gene Ontology ID 0008380 with the genes listed in the CellAge database. The trends of expression (over- or under-expression) associated with aging for these genes, as well as the effects (induction or inhibition) of these changes on the senescent phenotype, are shown.

Gene Symbol	Expression	Senescence Effect
AHNAK2	Over-expressed	
RBM24	Over-expressed	
ALYREF	Under-expressed	
BUD13	Under-expressed	
C1QBP	Under-expressed	
CWF19L1	Under-expressed	
DAZAP1	Under-expressed	
DCPS	Under-expressed	
DDX39A	Under-expressed	
DHX15	Under-expressed	
EIF4A3	Under-expressed	
FUS	Under-expressed	
GEMIN6	Under-expressed	
HNRNPA1	Under-expressed	Inhibits
HNRNPA2B1	Under-expressed	
HNRNPA3	Under-expressed	Inhibits
HNRNPC	Under-expressed	Induces
HNRNPF	Under-expressed	
HNRNPH1	Under-expressed	
HNRNPH3	Under-expressed	
HNRNPM	Under-expressed	
HNRNPU	Under-expressed	
KHDRBS1	Under-expressed	
KHSRP	Under-expressed	
LSM2	Under-expressed	
LSM3	Under-expressed	
LSM5	Under-expressed	
LSM6	Under-expressed	
NPM1	Under-expressed	Inhibits
PRPF38A	Under-expressed	
PRPF4	Under-expressed	
PTBP1	Under-expressed	
RBMX	Under-expressed	
RSRC1	Under-expressed	
SF3B3	Under-expressed	
SFPQ	Under-expressed	
SNRNP40	Under-expressed	
SNRPA	Under-expressed	
SNRPB	Under-expressed	
SNRPB2	Under-expressed	
SNRPC	Under-expressed	
SNRPD1	Under-expressed	
SNRPE	Under-expressed	
SNRPF	Under-expressed	
SRSF3	Under-expressed	
SRSF7	Under-expressed	
SYNCRIP	Under-expressed	
THOC1	Under-expressed	
TRA2B	Under-expressed	
TSEN15	Under-expressed	

**Table 2:** Impact of compounds and molecules on splicing events, splicing factors, and aging.

Compound/Molecule	Action on Splicing Events	Effects on Splicing Factors	Potential Anti-Aging or Pro-Aging
**Antisense Oligonucleotides (ASOs)**	Correct mRNA mis-splicing	May restore normal splicing factor levels	Potential Anti-Aging (e.g., LMNA mutation correction)
**Pladienolide B**	Inhibits spliceosome	May alter splicing patterns	Effects on aging not fully understood
**Spliceostatin A**	Inhibits spliceosome	Alters splicing patterns	Effects on aging not fully understood
**E7107**	Inhibits spliceosome	Induces senescence in certain contexts	Context-dependent effects on aging
**Hexanucleotide GGGGCC ASOs**	Target repeat-containing C9ORF72 RNA	Decrease repeat-containing RNA variants, relieves age-dependent deficits	Potential Anti-Aging (in ALS/FTD with C9ORF72 repeat expansion)
**Exosome-targeting drugs**	Promote degradation of transcripts with retained introns	May remove senescent RNA transcripts and splicing factors	Potential Anti-Aging (by removing senescent cells)

**Table 3:** Possible criteria and key factors for identifying a splicing factor or event as a “driver” of the aging process.

Criteria	Description
**Causality and Temporality**	Establish a causal relationship, demonstrating that changes in the factor or event precede or coincide with aging-related changes.
**Functional Consequences**	Investigate the functional consequences, especially regarding splicing patterns of genes involved in aging-related processes.
**Consistency Across Tissues and Species**	Observe consistent alterations in multiple tissues and across species during aging.
**Impact on Health and Longevity**	Assess associations with age-related health outcomes, disease susceptibility, and overall longevity.
**Genetic and Epigenetic Regulation**	Explore genetic mutations or epigenetic modifications linked to changes in the splicing factor or event during aging.
**Relevance to Age-Related Diseases**	Examine its contribution to age-related diseases, indicating a role in aging.
**Biological Mechanisms**	Investigate its impact on cellular processes, signaling pathways, and molecular pathways associated with aging.
**Modification by Interventions**	Determine if interventions can modulate the factor or event, influencing aging.
**Longitudinal Studies**	Track changes over an individual’s or organism’s lifespan to understand its evolution with age.
**Population Variability**	Consider variability across different individuals and populations.
**Comparisons of phenotype profiles “Young”/”Aged”**	Compare profiles in aged individuals to those of younger individuals.
**Contribution to Hallmarks of Aging**	Evaluate its contribution to recognized hallmarks of aging.
